# Pictorial History of the War for the Union

**Published:** 1863-11

**Authors:** 


*Pictorial History of the War for the Union.*

We have received the first volume of a work entitled *Pictorial History of the War for the Union,* embracing a complete and reliable history of the war from its commencement; giving a graphic picture of its encounters, thrilling incidents, frightful scenes, hairbreadth escapes, individual daring, desperate charges, personal anecdotes, etc., gleaned from eye-witnesses of, and participants in the terrible scenes described—a truthful living reflex of all matters of interest connected with the most gigantic of human struggles; together with a complete Chronological Record, giving every event in the order of its occurrence. W. D. Griffing is the agent for this valuable work and no other opportunity will be offered to obtain it except of him.

